# Comparison of postoperative isokinetic quadriceps and gluteal muscular strength after primary THA: is there an early benefit through enhanced recovery programs?

**DOI:** 10.1186/s40634-023-00687-9

**Published:** 2023-11-22

**Authors:** Jan Reinhard, Annika Schreiner, Silvia Dullien, Julia Sabrina Götz, Franziska Leiss, Günther Maderbacher, Joachim Grifka, Felix Greimel

**Affiliations:** Department of Orthopedics, University Medical Center Regensburg, Asklepios Klinikum Bad Abbach, Kaiser-Karl-V-Allee 3, 93077 Bad Abbach, Germany

**Keywords:** Total hip arthroplasty (THA), Fast track surgery, Early mobilization, Enhanced recovery after surgery (ERAS), Biodex, Isokinetic strength measurement

## Abstract

**Purpose:**

Although total hip arthroplasty (THA) is expected to result in a postoperative loss of muscular strength, no study investigated the benefit of an enhanced-recovery-after-surgery (ERAS) concept on the hip muscles in detail. We evaluated if (1) an ERAS-concept for primary THA results in reduced loss of muscular strength five days and four weeks postoperative. We (2) compared the two groups regarding Patient-Related-Outcome-Measures (PROMs), WOMAC-index (Western-Ontario-and-McMaster-Universities-Osteoarthritis-Index), HHS (Harris-Hip-Score) and EQ-5d-3L-score.

**Methods:**

In a prospective, single-blinded, randomized controlled trial, we compared isokinetic muscular strength of 24 patients receiving primary THA with an enhanced recovery concept with early mobilization (*n* = 12, ERAS-group) and such receiving conventional THA (*n* = 12, non-ERAS). Isokinetic muscular strength was measured with a Biodex-Dynamometer before, as well as five days and four weeks after surgery (peak-torque, total-work, power). Furthermore, WOMAC, HHS, PROMs and EQ-5d-3L were imposed.

**Results:**

The ERAS group revealed significant higher isokinetic strength (peak-torque, total-work, power) at both time points. Both groups showed a significant pain decrease at both time points meeting very high rates of patient satisfaction resembled by good results in PROMs, WOMAC, HHS, EQ-5d. There was no significant difference in any of the scores between both groups.

**Conclusion:**

We proved a significant reduced loss of muscular strength five days and four weeks after primary THA in combination with an ERAS concept. However, the reduced loss of muscular strength is not reflected by patient’s functional outcome and quality of life, showing no significant differences in WOMAC, HHS, EQ-5d-3L, PROMs and NRS. Therefore, this study supports the implementation of an ERAS concept for primary THA in terms of isokinetic strength. Further studies are needed to evaluate the development of muscular strength over a long period.

## Introduction

Enhanced recovery after surgery (ERAS) leads to less morbidity through faster recovery and therefore shorter hospital stays [15, 22, 27]. Growing acceptance and worldwide adoption of ERAS concepts recently lead to an access in orthopedic surgery [14, 16, 25]. Primary total hip arthroplasty (THA) requires a long recovery and is accompanied by resulting pathophysiologic catabolism. ERAS concepts aim to counteract this side effect by early mobilization. Recent studies prove its efficacy with less thromboembolic and gastrointestinal adverse reactions [2, 16, 17]. Primary THA in the U.S. is expected to increase, reaching 1.429.000 surgical interventions annually in 2040, illustrating a percent rise of 284% [26]. In 2007, THA was voted the most successful operation of the century [19]. Nevertheless, almost 10% of patients complain postoperative dissatisfaction [5, 12, 13]. Stabilization of the pelvis on frontal plane is mainly accomplished by the *gluteus medius* and *minimus* muscles [18]. Insufficiency of these two muscles may be a main reason for persistent dissatisfaction after primary THA [6]. Isokinetic dynamometers as the Biodex system, represent the gold standard for assessment of muscular strength [8]. While isokinetic measurement for the knee is well investigated and established, to date isokinetic strength measurement of the hip is rarely performed and strong reference values are still missing [10, 34]. In colorectal surgeries, which ERAS concepts were initially developed for, reduced loss of muscular strength is described [16]. Although THA is expected to lead to a loss of muscular strength, no study investigated isokinetic strength after primary THA with an ERAS concept and conventional THA at such early time point in detail.

### Aim of the study

In a prospective randomized controlled study, we aimed to evaluate if (1) an ERAS-Concept leads to reduced loss of muscular strength five days and four weeks postoperative in comparison to conventional THA. Furthermore, we (2) compared the two groups regarding the patient-related outcome measures (PROMs), the WOMAC index (Western Ontario and McMaster Universities Osteoarthritis Index), the HHS (Harris hip score) and the EQ-5D-5L.

## Methods

Data assessment was conducted between April 2021 and January 2022 at a tertiary reference center and maximum provider for arthroplasty. Altogether, 31 patients were included in this prospective, single-blinded, randomized controlled trial. Surgery was performed by three senior orthopedic surgeons. Except the surgeons, who only performed the operation and were not involved in the follow-up, every patient as well as every member of the study crew were blinded. To prevent unblinding of patients, each group was treated on different wards postoperatively to avoid contact between groups.

Main criterion for inclusion was primary or secondary osteoarthritis of the hip with indication for primary THA. Criteria for exclusion met walking distance less than 100 m, use of rollator / wheelchair, earlier surgical interventions to the hip, body mass index (BMI) > 40 kg/m^2^, tumor disease or pronounced muscular contractures. Furthermore, patients were excluded who were younger than 18 or above 90 years, participated in a different study or refused to participate. During the consultation-hour, patients that met the inclusion and exclusion criteria were informed orally and in written form about the study and asked if they would like to participate. The informed consent was signed before enrollment by every participant. Participation was voluntary with withdrawal possibility at any time. All enclosed patients were evenly distributed on both groups using closed envelopes. The present study is part of a big single-blinded randomized controlled trial, comparing an ERAS and Non-ERAS group for primary THA. It was conducted in agreement with the ethical standards of the Declaration of Helsinki (1975). The study was approved by the local Ethics Committee (approval number 19–1308-101). The registration number in the DRKS is called DRKS00031345 (WHO register).

Patients in both groups received primary THA via a modified Watson-Jones approach without transection of muscular tissue [4]. Patients were placed in lateral position and an anterolateral mini-incision was performed. Using the intermuscular plane between *Musculus tensor fasciae lata* and *Musculus gluteus medius*, the integrity of the muscles is preserved while the intactness of the posterior capsule prevents posterior dislocation [4]. Each patient received the collarless, cementless CORAIL® stem, a PINNACLE® acetabular cup, an ALTRX® polyethylene liner and depending on age and allergies either a BIOLOX® delta ceramic head or a metal head (DePuy Synthes, Raynham, MA, U. S.).

Each patient in the enhanced recovery after surgery group (ERAS group) received preoperative gait training with crutches and detailed education about pain management and the precautions after THA. Directly before the operation, patients received a single dose of non-steroid-anti-inflammatory analgesia (etoricoxibe 90 mg). Surgical intervention was performed under short-lasting spinal anesthesia (4 ml prilocaine 1%, hyperbaric and 10 µg sufentanil) and administration of dexamethasone (8 mg) intravenously. Intraoperatively, tranexamic acid was applied systemically (1 g) and topically (2 g) as well as local-infiltration analgesia (ropivacaine 200 mg, adrenaline 0.5 mg). No drains were inserted. Patients in ERAS group were instantly allowed full weight-bearing and were first mobilized two to three hours after the intervention. A walking distance of 50 m was aimed for first mobilization. A physiotherapy treatment protocol for enhanced recovery after THA was developed. Targeted physiotherapy was performed twice a day by two physiotherapists, specifically trained in terms of ERAS concepts. Physical therapy consisted of mobilization and muscular strengthening but also thrombosis and pneumonia prevention. To autonomously improve muscular strength of the hip, the patients were instructed to work out on a specifically created exercise circuit, containing different workouts for muscle formation, a walking course and coordination tasks. Postoperatively patients received a special pain management concept, based upon World health organization (WHO) three step analgesic ladder [8]. Both groups received Ibuprofen three times and Metamizole four times a day. The first three days postoperatively every patient within the ERAS group received 10/4 mg oxycodone/naloxone once a day in addition. The pain medication on demand was identical in both groups. Administration depended on the NRS. On intermediate care unit, patients received 3 mg piritramide optionally. On the ward, patients received 100 mg tramadol or 10/5 mg oxycodone/naloxone as additional operational analgesics.

Patients who received conventional THA (non-ERAS) neither received special education nor single dose analgesics preoperatively. Anesthesia was maintained by a long-lasting spinal anesthesia (4 ml bupivacaine, 0.5% and fentanil). Neither tranexamic acid nor local infiltration analgesia was administered intraoperatively. In all cases wound drains were inserted. Postoperatively, patients were allowed full weightbearing and they were mobilized for the first time on the first day after operation. Physiotherapy was only performed once a day and they did not use the exercise circuit. See Table 1 for a brief comparison of both concepts.

**Table 1:** Overview on the comparison of the two concepts for primary THA

	**Non-ERAS (*n* = 12)**	**ERAS (*n* = 12)**
**Preoperatively**		
Gait training with crutches	**-**	** + **
Patient education	**-**	** + **
Etoricoxibe 90 mg p.o. preoperatively	**-**	** + **
**Intraoperatively**		
DePuy Synthes CORAIL® cementless stem, PINNACLE press fit cup	** + **	** + **
Short lasting spinal anesthesia (Prilocain 1%, 10 µg sufentanil, Dexamethasone 8 mg i.v)	**-**	** + **
Long-lasting spinal anesthesia (4 ml bupivacaine 0.5% and fentanil)	** + **	**-**
Local infiltration analgesia (Periacetabular, femoral, subcutaneously)	**-**	** + **
Tranexamic acid local and topically	**-**	** + **
Drains	** + **	**-**
**Postoperatively**		
First mobilization	1 d postoperatively	2–3 h postoperatively
Full weight bearing	** + **	** + **
Physiotherapy	1x/d	2x/d
Exercise circuit	**-**	** + **

Patients in both groups were discharged to the rehabilitation clinic five to seven days after the surgery took place. After initial treatment at our hospital, they stayed in the rehabilitation clinic for another three weeks. This is common in Germany because of the general laws in the German health system (SGBV). The time points for readmission were chosen on behalf of the discharge from the acute and the rehabilitation clinic. Therefore, the first time point was chosen with the objective that all patients were still in the operating hospital, and it was less stressful for the patients to perform the measurements. Moreover, at five days postoperatively, one can assume that all patients were successfully mobilized and could already walk on their own. The second time point was chosen directly after the rehabilitation clinic. Patients of both groups received the same therapy in the rehabilitation clinic for three weeks. By discharge they usually return to daily routine.

### Clinical examination, PROMs, WOMAC, HHS, EQ-5D-3L

Within every consultation, patients were asked about pain intensity on each side, quantified by a numeric rating scale (NRS) ranging from zero (no pain) to ten (maximum pain). Furthermore, we measured maximum passive flexion and abduction of the operated hip. Each patient was tested for Trendelenburg’s sign [31] and the possibility of one-leg standing on the injured side for more than 15 s. A special questionnaire was used to analyze postoperative satisfaction and quality of life. We set up seven questions: (1) How do you rate the function of your hip? (normal / almost normal / unnormal / strongly unnormal). (2) Do you judge the operation as successful (yes / no)? (3) Would you undergo the operation (THA) again (yes/no)? (4) Have your expectations to the operation been fulfilled? (no / light / moderate / strong / very strong)? (5) How do you feel in comparison to preoperative health condition (much better / better / same / worse / much worse)? (6) Has your quality of life improved (no / light / moderate / strong / very strong)? Moreover, WOMAC-Index, HHS, as well as EQ-5D-3L were imposed. Both, WOMAC and HHS are popular and validated scoring tools. The WOMAC score is composed of pain (0 – 20 points), stiffness (0 – 8 points) and physical function (0 – 68 points). The best result for the HHS is 100 points, while 60 to 69 points resemble a bad result. EQ-5D-3L scores the five dimensions of health-related quality of live. It ranks from one to three points, resembling none to extreme problems [20].

### Isokinetic strength measurement

Identically in both groups, each patient obtained isokinetic muscular strength measurement within one week before operation as well as five days and four weeks after operation for each hip joint separately. For isokinetic strength measurements we used a *Biodex System 4 Pro Dynamometer* (Biodex Medical systems, Shirley, NY, U. S.). To achieve optimal and reproducible results the test protocol was set up in close contact with the manufacturer Biodex Medical systems. The machine was maintained and calibrated by a special mechanic of the company prior to the final study. A modified validated test protocol, provided by the manufacturer was used. All measurements were performed by two blinded observers. To achieve an experimental setup as close as possible to physiological motion in daily life we chose an upright position of the patient, accepting a possible reduction of isokinetic strength on the healthy side due to required one leg stand on the operated side. For every patient the Biodex Dynamometer was adjusted individually for height and length of the femur before the first measurement. The superior apex of the greater trochanter was identified, and the rotation center of the dynamometer was set two centimeter above its heigh. The lever length was adjusted so that the femoral pad ended 15 cm above the patella. If patients’ height measured less than 160 cm, a foot stool of 13 cm height was used. The parameters, defined at the beginning, were maintained over the whole study to achieve comparable results. Before every measurement the dynamometer performed a self-calibration.

We sought to evaluate the most important hip moving directions for daily life with an artificial hip joint: Extension / Flexion and Abduction / Adduction. Before each measurement, patients were required to walk a fixed distance of 100 m to warm up. In addition, they performed five contractions in exercise mode before the final measurements. The healthy leg was always measured first, followed by the injured side. SeeFig. 1*.*

**Fig. 1:**
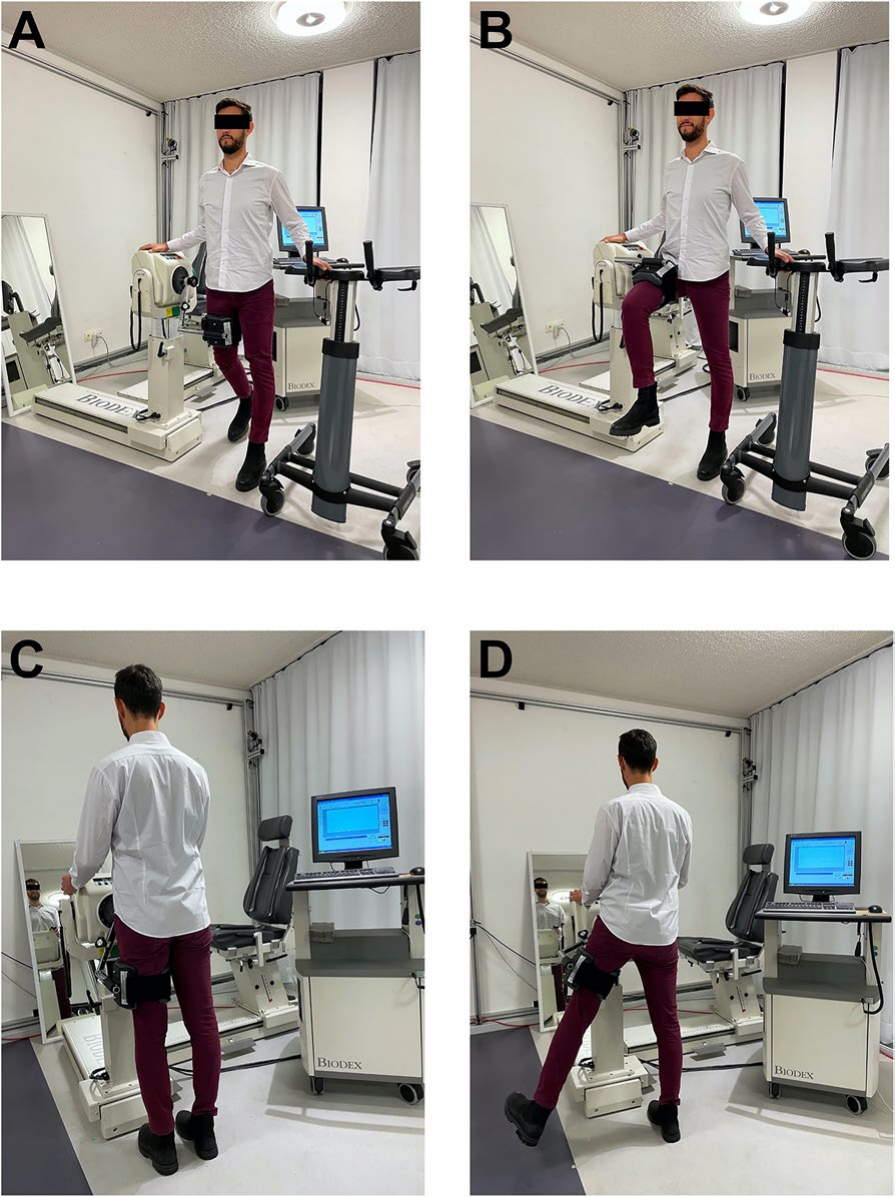
Execution of isokinetic strength measurement with the Biodex system. Execution of isokinetic strength measurement in a special gait laboratory at our tertiary reference center. **A** and **B** show the measurement of Flexion and Extension. Picture **C** and **D** demonstrate the Adduction and Abduction of the hip

Isokinetic strength measurement of Extension / Flexion was executed with an angular speed of 60°/s. The patient was placed beside the dynamometer and the femur was attached to the lever after the individual height was chosen. Patients were told to hold up on tested side on the Biodex machine and on opposite side on a walker, individually adapted to patients’ height. First the individual range of motion was defined by maximal flexion and maximal extension. Afterwards patients were told to perform three practice runs with maximum strength, using the full range of motion, defined before. The final measurement was done with maximum strength and five repetitions. Patients were cheered up and motivated to achieve and maintain their maximum strength. A study member always controlled if the movement was carried out in a clear and fluent way with straight back and no evasive movement, if not the whole measurement was repeated.

Isokinetic strength measurement of Abduction / Adduction was done with an angular speed of 30°/s. The patient was placed facing the dynamometer and attached to the lever, height was not changed in comparison to Extension / Flexion. Patients were told to hold up on each side of the Biodex machine. A mirror placed opposite of the patient guaranteed clear movement and control of the patients’ movements. A team of the study crew made sure patients do not tilt heir pelvis or do outer rotation with the leg. The individual range of motion was set for Abduction only, the Adduction was set as 0° on behalf of the precautions after THA. The cycle was identical as described in Extension / Flexion. See attached pictures of the measurement set up for maximum Extension / Flexion and Abduction / Adduction. See Fig. 1.

For extension / flexion and abduction / adduction we imposed the peak torque (measured in Newton meter, NM), the overall work (measured in Joule, J) and the overall power (measured in Watt, W). The Biodex system calculates the variation coefficient, which resembles the reproducibility of each movement. Furthermore, the Biodex system measures the active range of motion of each moving direction (Extension / Flexion and Abduction / Adduction).

### Statistical analysis

The Shapiro–Wilk-Normality-Test was used to test for normal distribution. Metric variables are noted as mean ± standard deviation (SD) if the data is normally distributed or as median ± interquartile range (IQR) if not. Categorical variables are noted in relative frequency. To test for statistical significance, we used *t-test* if data was normally distributed, or *nonparametric Mann–Whitney U*. To test for variance homogeneity, we used one factor ANOVA with post-hoc Tukey test.

Statistical significance was considered *p* < 0.05. Statistical analysis was performed with SPSS (IBM SPSS Statistics 28, International Business Machines Corporation (IBM), Armonk, New York, U.S.).

## Results

**Fig. 2:**
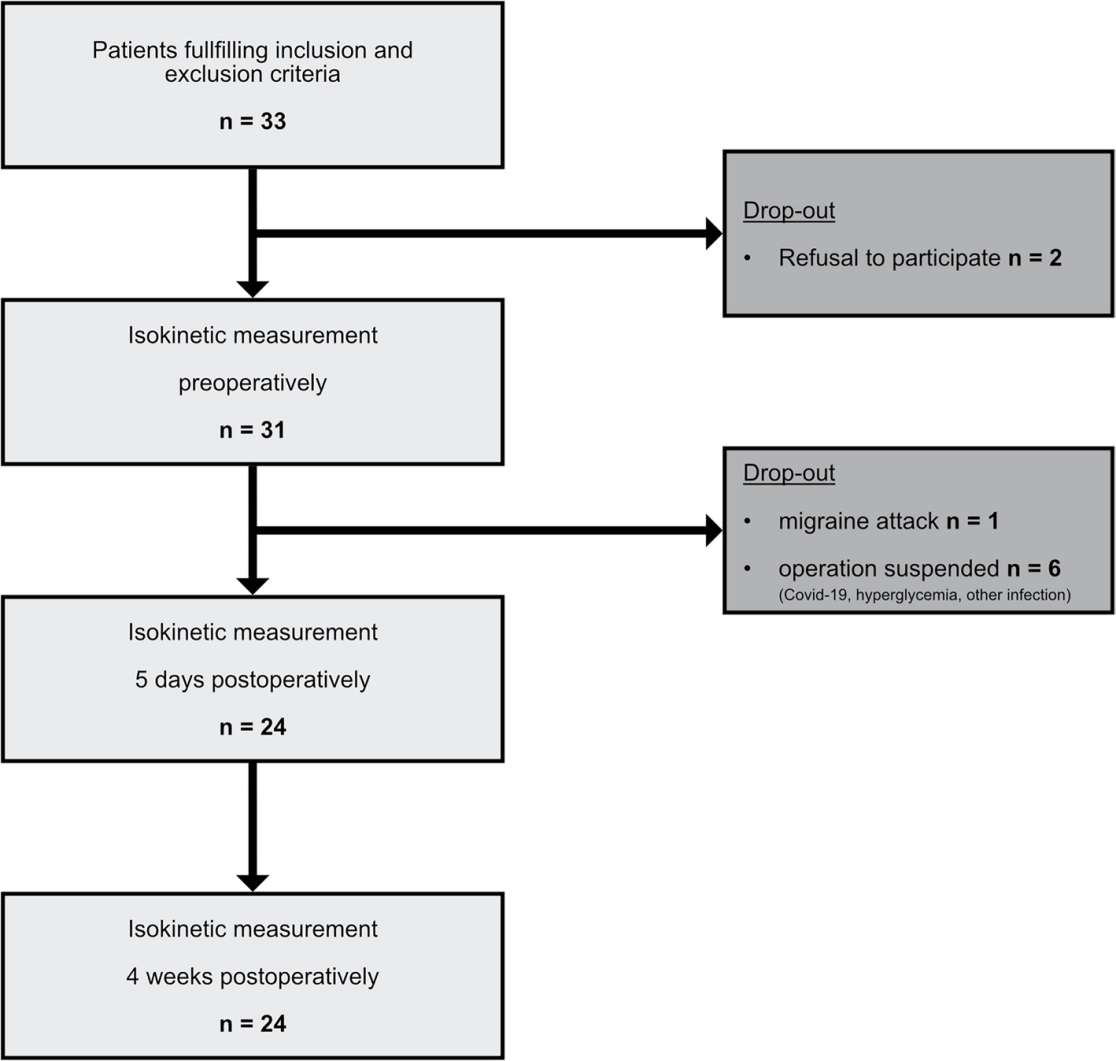
Flowchart methods. Flowchart of the enrollment process and the follow-up. Initial patient population and resulting patients after drop-out

Between 04/2021 and 01/2022, 33 patients were enrolled during consultation hour. Afterwards two patients revoked participation. The preoperative isokinetic measurement was performed in 31 patients. In six cases, surgery was delayed for indefinitely: Three patients were diagnosed with a SARS-Covid-19 infection preoperatively, one case showed unknown hyperglycemia requiring an amendment and in two patients high inflammation values requiring further diagnostic were detected in preoperative laboratory testing. One patient suffered from a severe migraine attack on ward and was not able to perform the measurements. Finally, twelve patients received primary THA with enhanced recovery after surgery (ERAS) and twelve patients conventional primary THA at our tertiary reference hospital. The flowchart is illustrated in Fig. 2.

### Demographic data

Analysis of demographic data revealed no significant differences between the two groups (*p* > *0.05*, see Table 2). Especially the parameters age, sex, Body-mass-index (BMI) and injured leg ranged among comparable values.

**Table 2:** Demographic data of the 24 included patients

	**Non-ERAS (*n *= 12)**				**ERAS (*n* = 12)**				**Statistical difference**
	Mean ± SD		Range		Mean ± SD		Range		*p* – value
Age (years)	68.19 ± 8.7		54 – 82		68.92 ± 10.61		51 – 87		*0.94*
Sex (male: female)	5: 7				6: 6				*1.0*
Body mass index (BMI)	28.03 ± 5.03		19.83 – 38.42		27.8 ± 4.18		22.04 – 36.76		*0.89*
Injured leg (right: left)	6: 6				6: 6				*1.0*
Dominant leg (right: left)	10: 2				12: 0				*0.48*
Osteoarthritis contralateral (mild / symptomatic)	2 / 1				4 / 0				*0.64*
Total hip arthroplasty (THA) contralateral	2				1				*1.0*
ASA Score frequency (%)	1	2	3	4	1	2	3	4	*0.14*
	8.3	83.3	8.3	0	33.3	58.3	8.3	0	
Duration of surgery (min)	57.25 ± 13.75		35 – 85		58.33 ± 12.14		36 – 77		*0.28*

## Pain development and functional outcome

**Fig. 3:**
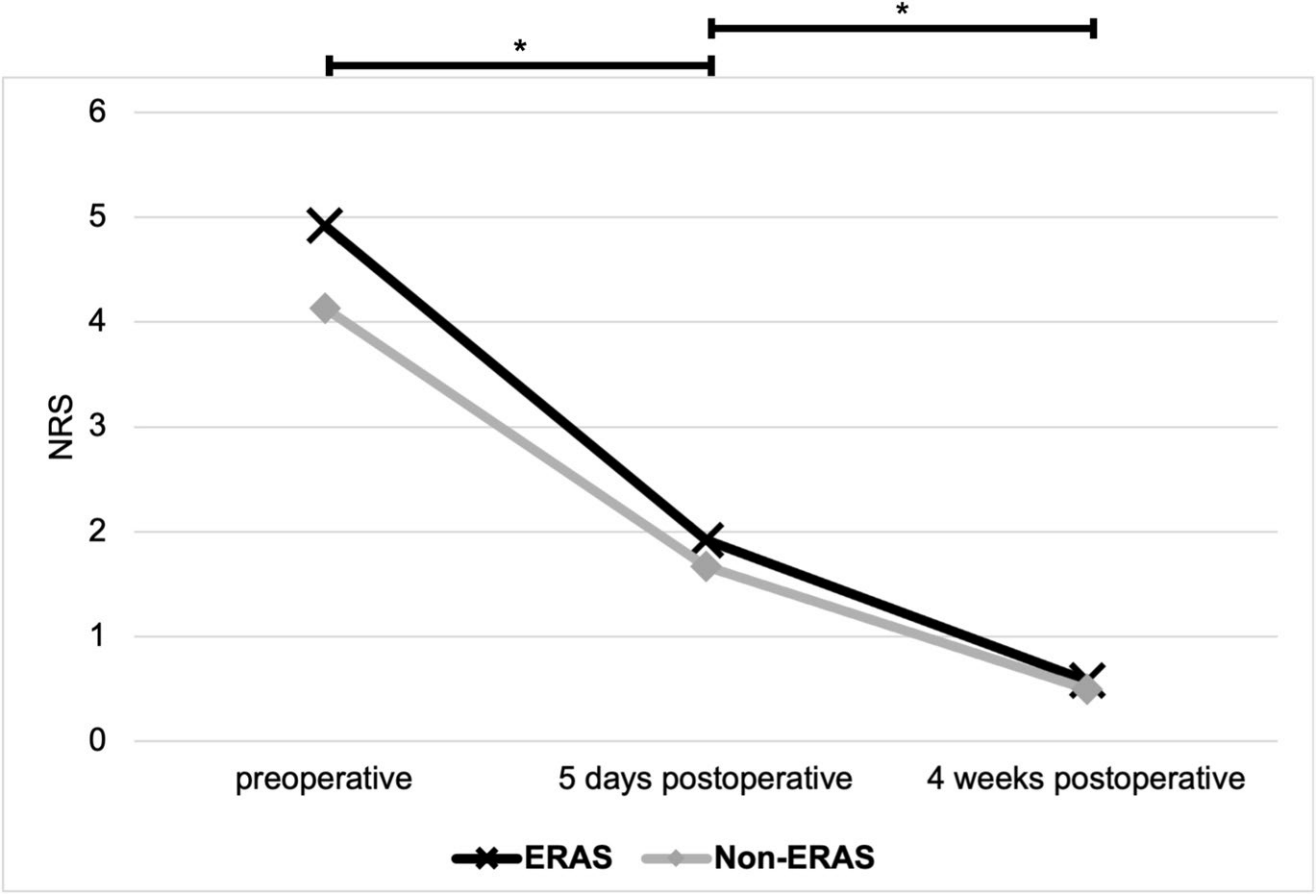
Pain development (NRS) on the operated hip. Demonstration of pain on the operated side, measured with an NRS. Comparison preoperative as well as five days and four weeks postoperative. The pain significantly decreased between the three time points (marked by *). There were no significant differences between both groups

The evaluation of pain development showed a significant decrease of pain on the operated side in both groups at every time point (all *p* < *0.05*). There were no significant differences between the two groups (*p* > *0.05*). The pain evaluation of the contralateral hip did not show significant differences. See Fig. 3*.*

In the ERAS group, one quarter of patients showed positive Trendelenburg’s sign preoperatively, decreasing to zero patients four weeks after THA. In contrast, we observed an increment of Trendelenburg’s sign five days postoperatively in the Non-ERAS group, decreasing four weeks after the operation. There were no significant differences between both groups at any of the three time points (all *p* > 0.05). See Table 3*.* In both groups around two-thirds of patients were able to perform a one-leg stand preoperatively (*p* > 0.05). In the non-ERAS group, the ability significantly decreased five days after THA (*p* = 0.02) and increased again, insignificantly. In the ERAS group, we detected lesser decline (*p* > 0.05). We could not detect significant differences between the two groups (*p* > 0.05). The evaluation of passive range of motion five days postoperative showed no significant differences in postoperative flexion. The range of motion of abduction was significant better in the ERAS group five days postoperatively (*p* < *0.001). *See Table 3*.*

**Table 3:** Positive Trendelenburg’s sign, ability to one-leg stand (> 15 s) and passive range of motion of the operated hip

		**Non-ERAS (*n* = 12)**	**ERAS (*n *= 12)**	***Statistical difference***
**Positive Trendelenburg’s sign (%)**				***p-value***
PRE-OP		16.7	25	*0.99*
5 d POST-OP		50	8.3	*0.07*
4 w POST-OP		8.3	0	*0.99*
**Possibility One-leg stand > 15 s (%)**				
PRE-OP		66.7	58.3	*0.99*
5 d POST-OP		16.7	50	*0.19*
4 w POST-OP		58.3	83.3	*0.37*
**Passive range of motion (°) mean ± SD**				
PRE-OP	Flexion	103.1 ± 14.9	95.5 ± 7.6	*0.36*
	Abduction	26.3 ± 5.2	21.5 ± 6.7	*0.12*
5 d POST-OP	Flexion	96.7 ± 6.6	98.8 ± 9.9	*0.7*
	Abduction	27.73 ± 6.8	37.5 ± 4.6	** < *0.001***
4 w POST-OP	Flexion	104 ± 12.7	109.2 ± 9.9	*0.28*
	Abduction	39 ± 7.4	39.2 ± 5.2	*0.83*

### Patient related outcome measures (PROMs)

The evaluation of patient related outcome measures proved very high rates of patient satisfaction in both groups. Some questions as health condition in comparison to preoperative situation showed a tendency to better results within the ERAS group, however we could not detect significant differences between both groups at five days as well as four weeks postoperatively (*p* > *0.05)*. At four weeks the results in both groups were almost identical. See Table 4.

**Table 4:** Results of Patient related outcome measures (PROMs) five days and four weeks postoperatively

**PROMS**		**Non-ERAS (*n* = 12)**		**ERAS (*n *= 12)**		***p-value***	
		5 days	4 weeks	5 days	4 weeks	5 days	4 weeks
How do you rate the function of your hip?	Normal	4/9	7/12	2/9	6/12	*0.62*	*0.99*
	almost normal	5/9	5/12	7/9	6/12		
	impaired	0/9	0/12	0/9	0/12		
	strongly impaired	0/9	0/12	0/9	0/12		
Do you judge the operation as successful?	yes	11/11	12/12	10/10	12/12	*0.99*	*0.99*
	no	0/11	0/12	0/10	0/12		
Would you undergo the operation (THA) again?	yes	11/11	12/12	10/10	12/12	*0.99*	*0.99*
	no	0/11	0/12	0/10	0/12		
Have your expectations to the operation been fulfilled?	very strong	7/11	8/12	6/10	7/12	*0.99*	*0.99*
	strong	4/11	3/12	4/10	5/12		
	moderate	0/11	1/12	0/10	0/12		
	light	0/11	0/12	0/10	0/12		
	no	0/11	0/12	0/10	0/12		
How do you feel in comparison to your preoperative health condition?	much better	6/11	9/12	9/10	9/12	*0.15*	*0.99*
	better	5/11	3/12	1/10	3/12		
	same	0/11	0/12	0/10	0/12		
	worse	0/11	0/12	0/10	0/12		
	much worse	0/11	0/12	0/10	0/12		
Has your quality of life improved?	very strong	2/11	7/12	4/10	4/12	*0.5*	*0.41*
	strong	7/11	4/12	4/10	8/12		
	moderate	1/11	1/12	1/10	0/12		
	light	1/11	0/12	1/10	0/12		
	no	0/11	0/12	0/10	0/12		

### WOMAC, HHS, EQ-5D-3L

The WOMAC-index and HHS revealed a significant improvement five days as well as four weeks postoperative. There were no significant differences between both groups at any time point. The same results were obtained in the evaluation of the EQ-5D-3L with improvement in every category. Analogous to the other scores there was no significant difference between both groups. See Table 5.

**Table 5:** Western Ontario and McMaster Universities Osteoarthritis Index (WOMAC), Harris hip score (HHS) and EQ-5D-3L—preoperative, five days and four weeks postoperatively

		**Non-ERAS (*n* = 12)**	**ERAS (*n* = 12)**	**Statistical difference**
**WOMAC (mean ± SD)**				*p*-value
Stiffness (0–8)	PRE-OP	3.3 ± 1.8	4.4 ± 1.2	*0.18*
	5 d POST-OP	2.2 ± 1.8	2.6 ± 2	*0.84*
	4 w POST-OP	0.9 ± 1.1	1.3 ± 1.4	*0.84*
Pain (0–20)	PRE-OP	8.8 ± 4.1	10.1 ± 3	*0.34*
	5 d POST-OP	3.1 ± 3.2	3.3 ± 4.6	*0.99*
	4 w POST-OP	1.1 ± 1.4	1.6 ± 2.7	*0.99*
Physical Function (0–68)	PRE-OP	28.7 ± 9.8	36.3 ± 8.8	*0.08*
	5 d POST-OP	13.9 ± 13.1	13.1 ± 15.6	*0.48*
	4 w POST-OP	4.3 ± 5.8	5.6 ± 11.3	*0.48*
Total score (0–96)	PRE-OP	40.8 ± 14.1	49.5 ± 14.1	*0.12*
	5 d POST-OP	14.8 ± 16.5	19 ± 20.8	*0.93*
	4 w POST-OP	6.3 ± 7.9	8.5 ± 14.3	*0.93*
**HHS (mean ± SD)**				
Pain (0–44)	PRE-OP	17.1 ± 4.9	6.4 ± 1.9	*0.55*
	5 d POST-OP	36.6 ± 7.9	30.8 ± 14.1	*0.45*
	4 w POST-OP	40.9 ± 4.1	42.4 ± 4.3	*0.45*
Walking (0–33)	PRE-OP	20.6 ± 8.8	23.1 ± 6.4	*0.67*
	5 d POST-OP	22.3 ± 3.9	24.5 ± 7.9	*0.31*
	4 w POST-OP	30.5 ± 2.8	32.2 ± 1.4	*0.31*
ADL (0–14)	PRE-OP	9.7 ± 2.6	7.8 ± 3	*0.14*
	5 d POST-OP	8.9 ± 1.8	9.5 ± 2.7	*0.8*
	4 w POST-OP	12.6 ± 2	12.6 ± 1.8	*0.8*
Total score (0–91)	PRE-OP	47.4 ± 9.8	47.3 ± 11.8	*0.99*
	5 d POST-OP	60 ± 10.5	58 ± 25.1	*0.93*
	4 w POST-OP	83.9 ± 7	87.2 ± 5.2	*0.47*
**EQ-5D-3L (mean ± SD)**				
Flexibility (1–3)	PRE-OP	1.8 ± 0.6	1.7 ± 0.5	*0.99*
	5 d POST-OP	1.2 ± 0.4	1.3 ± 0.5	*0.64*
	4 w POST-OP	1 ± 0	1.1 ± 0.3	*0.99*
self-supply (1–3)	PRE-OP	1.2 ± 0.6	1.2 ± 0.4	*0.99*
	5 d POST-OP	1.1 ± 0.3	1.1 ± 0.3	*0.99*
	4 w POST-OP	1 ± 0	1.1 ± 0.3	*0.99*
General tasks (1–3)	PRE-OP	1.7 ± 0.7	1.8 ± 0.4	*0.51*
	5 d POST-OP	1.5 ± 0.7	1.4 ± 0.5	*0.99*
	4 w POST-OP	1 ± 0	1.1 ± 0.3	*0.99*
Pain (1–3)	PRE-OP	2.1 ± 0.3	2.2 ± 0.4	*0.99*
	5 d POST-OP	1.3 ± 0.5	1.5 ± 0.7	*0.53*
	4 w POST-OP	1.2 ± 0.4	1.2 ± 0.4	*0.99*
Anxiety (1–3)	PRE-OP	1.3 ± 0.5	1.1 ± 0.3	*0.31*
	5 d POST-OP	1.3 ± 0.5	1 ± 0	*0.21*
	4 w POST-OP	1 ± 0	1.1 ± 0.3	*0.99*

### Isokinetic strength measurement

**Fig. 4:**
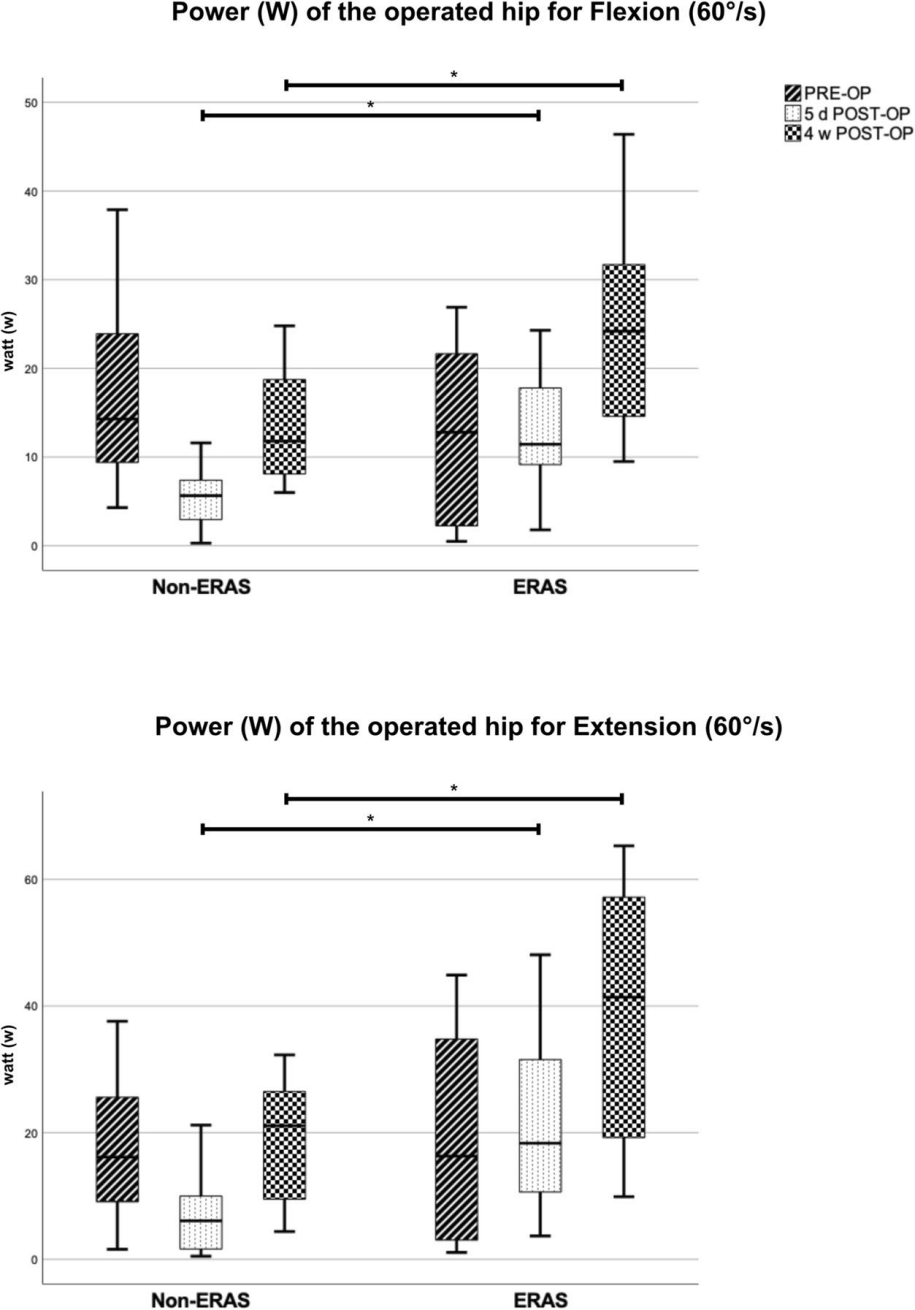
Isokinetic strength measurement – Power (W) analysis for Flexion and Extension (60°/s) on the operated hip, Boxplot, Significant differences between the two groups are marked by *

**Fig. 5:**
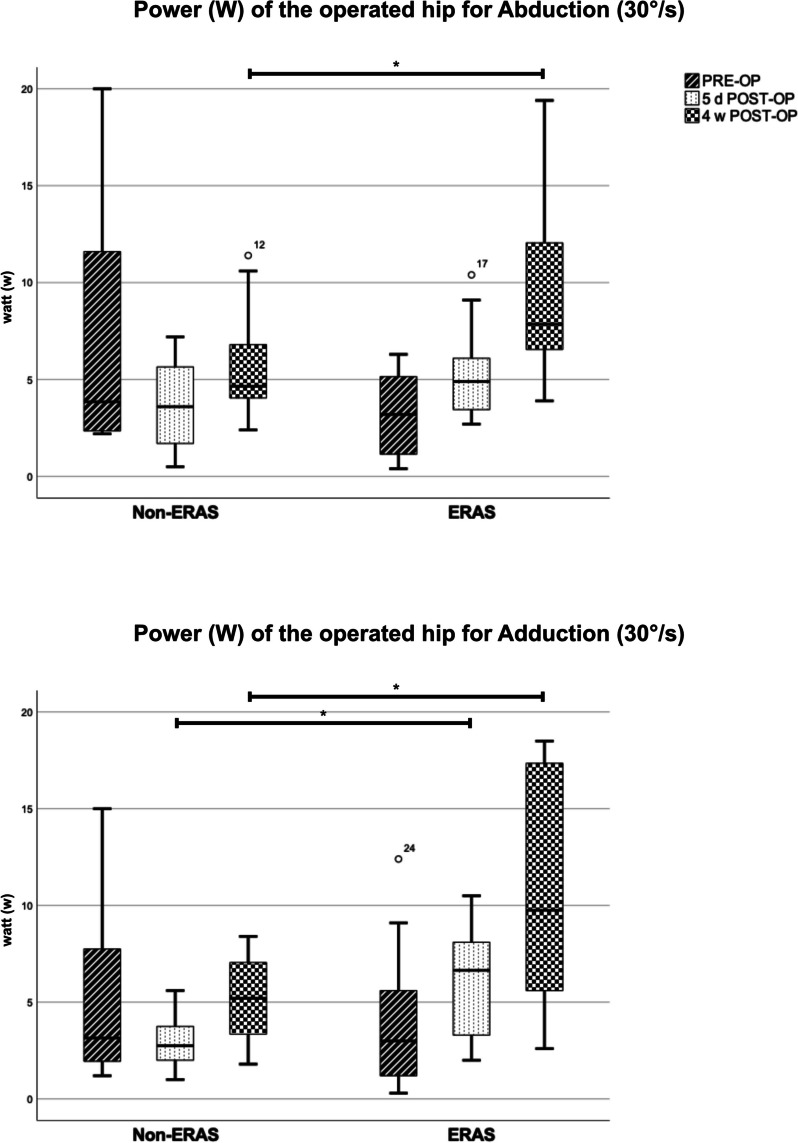
Isokinetic strength measurement – Power (W) analysis for Abduction and Adduction (30°/s) on the operated hip, Boxplot. Significant differences between the two groups are marked by *

The preoperative comparison between the two groups did not show any significant differences. Overall evaluation of isokinetic strength measurement of the operated hip showed significant better results in the ERAS group (see Table 6). In comparison to Non-ERAS group, patients demonstrated significant superior peak torque, overall work as well as power, for flexion, extension, and adduction five days as well as four weeks postoperatively. While patients in the Non-ERAS group mostly suffered from a strength reduction five days postoperatively, in patients of the ERAS group this was less pronounced, or they even showed a slight improvement of strength parameters (see Fig. 4 and 5). Isokinetic strength measurement of abduction showed superior results in the ERAS group concerning all parameters four weeks after the operation. Compared to the Non-ERAS group patients showed significant higher total work, and a tendency towards higher peak torque and power five days after the operation. The evaluation of the active range of motion measured by the Biodex system revealed significant higher degrees for Extension / Flexion and Abduction / Adduction five days postoperatively in the ERAS group (*p* < *0.05).* The range of motion for Extension / Flexion even showed significant higher values in the ERAS group four weeks postoperatively (*p* < *0.001)*. However, the ERAS group showed a tendency to lower degrees of range of motion preoperatively, significant for Abduction / Adduction (*p* = *0.028). *See Table 6*.*

**Table 6:** Results of isokinetic strength measurement of the operated hip

Peak torque		Non-ERAS (*n* = 12)	ERAS (*n* = 12)	Statistical difference
**(Nm), mean ± SD**				*p-value*
60°/s flexion	PRE-OP	38.63 ± 18.53	29.13 ± 17.51	*0.21*
	5 d POST-OP	17.98 ± 7.88	33.22 ± 15.45	***0.008***
	4 w POST-OP	30.35 ± 11.64	51.23 ± 21.55	***0.009***
60°/s extension	PRE-OP	40.22 ± 18.87	41.01 ± 29.67	*0.94*
	5 d POST-OP	21.88 ± 15.81	42.67 ± 21.51	***0.013***
	4 w POST-OP	41.49 ± 17.81	71.64 ± 33.97	***0.015***
30°/s abduction	PRE-OP	33.08 ± 18.92	19.06 ± 9.47	*0.16*
	5 d POST-OP	20.81 ± 11.36	25.19 ± 8.54	*0.3*
	4 w POST-OP	26.6 ± 11.63	38.65 ± 14.5	***0.035***
30°/s adduction	PRE-OP	25.42 ± 15.79	20.52 ± 14.45	*0.48*
	5 d POST-OP	16.13 ± 6.8	27.81 ± 11.64	***0.008***
	4 w POST-OP	24.72 ± 10.65	39.9 ± 17.77	***0.021***
**Overall Work (J)**				
60°/s flexion	PRE-OP	144.51 ± 97.26	108.38 ± 85.25	*0.34*
	5 d POST-OP	36.11 ± 24.11	103.87 ± 57.12	***0.002***
	4 w POST-OP	101.11 ± 59.09	230.04 ± 116.05	***0.003***
60°/s extension	PRE-OP	179.43 ± 104.25	171.13 ± 157.67	*0.99*
	5 d POST-OP	55.76 ± 55.95	198.01 ± 144.21	***0.001***
	4 w POST-OP	161.94 ± 94.59	399.83 ± 222.76	***0.001***
30°/s abduction	PRE-OP	49.56 ± 43.71	21.13 ± 15.94	*0.11*
	5 d POST-OP	21.28 ± 14.14	35.28 ± 16.19	***0.034***
	4 w POST-OP	40.35 ± 21.47	70.83 ± 35.79	***0.019***
30°/s adduction	PRE-OP	39.18 ± 32.45	28.44 ± 29.44	*0.24*
	5 d POST-OP	17.72 ± 10.01	42.54 ± 20.36	***0.002***
	4 w POST-OP	36.85 ± 19.41	85.24 ± 51.03	***0.008***
**Power (Watt)**				
60°/s flexion	PRE-OP	16.32 ± 9.78	13.44 ± 10	*0.48*
	5 d POST-OP	5.36 ± 3.3	12.5 ± 6.4	***0.002***
	4 w POST-OP	13.22 ± 6.31	23.76 ± 11.29	***0.012***
60°/s extension	PRE-OP	17.34 ± 10.38	18.8 ± 16.74	*0.99*
	5 d POST-OP	6.9 ± 6.07	21.22 ± 14.24	***0.003***
	4 w POST-OP	19.04 ± 9.32	38.46 ± 21.19	***0.002***
30°/s abduction	PRE-OP	7.06 ± 6.02	3.13 ± 2.12	*0.11*
	5 d POST-OP	3.69 ± 2.18	5.36 ± 2.39	*0.09*
	4 w POST-OP	5.71 ± 2.77	9.28 ± 4.33	***0.006***
30°/s adduction	PRE-OP	4.98 ± 4.19	3.93 ± 3.78	*0.45*
	5 d POST-OP	2.92 ± 1.36	6.08 ± 2.97	***0.004***
	4 w POST-OP	5.14 ± 2.25	10.64 ± 5.91	***0.009***
**Variation coefficient**				
60°/s flexion	PRE-OP	27.77 ± 17.59	33.72 ± 26.97	*0.53*
	5 d POST-OP	41.55 ± 13.13	31.58 ± 15.58	*0.1*
	4 w POST-OP	19.81 ± 9.61	22.73 ± 10.66	*0.49*
60°/s extension	PRE-OP	23.33 ± 19.4	28.48 ± 25.37	*0.93*
	5 d POST-OP	24.83 ± 9.06	11.19 ± 5.86	** < *0.001***
	4 w POST-OP	17.82 ± 10.32	10.49 ± 7.06	*0.05*
30°/s abduction	PRE-OP	27.56 ± 18.44	29.27 ± 19.02	*0.89*
	5 d POST-OP	28.28 ± 20.83	23.35 ± 10.41	*0.93*
	4 w POST-OP	17.86 ± 8	15.23 ± 6.66	*0.99*
30°/s adduction	PRE-OP	22.17 ± 13.81	18.38 ± 10.94	*0.47*
	5 d POST-OP	24.73 ± 20.57	13.78 ± 4.65	*0.18*
	4 w POST-OP	18.63 ± 10.32	10.98 ± 5.74	***0.045***
**Active range of motion (°)**				
Flexion / Extension	PRE-OP	94.23 ± 19.29	83.89 ± 28.01	*0.30*
	5 d POST-OP	61.24 ± 16.64	88.06 ± 15.8	***0.001***
	4 w POST-OP	77.83 ± 13.1	106.21 ± 13.13	** < *0.001***
Abduction / Adduction	PRE-OP	36.23 ± 7.74	27.53 ± 10.22	***0.028***
	5 d POST-OP	27.12 ± 4.62	33.72 ± 5.79	***0.005***
	4 w POST-OP	35.33 ± 6.02	40.46 ± 7.52	*0.08*

The isokinetic strength measurement of the healthy, not operated hip revealed no significant differences between both groups in terms of Extension / Flexion as well as Abduction / Adduction (*p* > *0.05)*. The comparison of extension strength of the healthy hip showed no difference in maximum peak torque and power. Because of the significant improved range of motion, we could observe a significant higher total work in the ERAS group four weeks postoperatively for this parameter.

The evaluation of the coefficient of variation showed a tendency towards lower values in the ERAS group. In comparison to the Non-ERAS group the values were significant lower after five days for extension and after four weeks for adduction. Inversely proportional to the significant strength decrease five days after the operation, we observed a tendency towards an increase of variation coefficient here. See Table 6*.*

## Discussion

In a prospective, single-blinded randomized controlled trial, we compared isokinetic strength measurement in patients who received primary THA in combination with an ERAS concept, and such who underwent conventional surgery and post-treatment. The most important finding represents the reduced loss of muscular strength in the ERAS group five days as well as four weeks postoperatively, indicated by significant higher peak torque, work, and power for flexion/extension as well as abduction/adduction (*p* < *0.05*). The study supports the use of an ERAS concept for primary THA, featuring early mobilization, local infiltration analgesia and intense postoperative physiotherapy. It is known that osteoarthritis of the hip leads to a loss of muscular strength of the affected side, accompanied by reduced muscular density [21, 24, 33]. Additionally, patients in need of primary THA represent a rather old cohort. In this study the mean age of patients was 68 years. Especially these patients are at risk for muscle breakdown due to longer immobilization. To prevent long immobilization and complications as pulmonary artery embolism, in particular the first days after surgery seem to be the most important ones. While the Non-ERAS group showed a distinct drop of strength five days after the operation, this dip was less pronounced in the ERAS group. The less distinct dip in terms of muscular strength five days postoperatively in the ERAS group leads to the assumption, that an ERAS concept successfully counteracts the postoperative catabolism and results in less muscle breakdown. Although, five days postoperatively there was no significant difference in terms of abduction, at four weeks’ time-point the patients of the ERAS group showed significant stronger abduction. This highlights the value of the ERAS concept used and illustrates that even though all patients received the same rehabilitation, they still benefit from the ERAS concept four weeks postoperatively.

However, the patient related outcome measures, WOMAC index, HHS, EQ-5d-3L as well as postoperative pain did not show significant differences between the two groups at either time point. This observation raises the assumption, that even though the short-term loss of muscular strength is significantly reduced, the difference seems not to be noticed by the patient himself. One reason may be the fact, that primary THA belongs to the most successful operations, meeting a patient satisfaction of over 90%. Our data is in line with the high rates of patient satisfaction, proved by WOMAC, HHS, EQ-5D-3L, PROMS and NRS. Bhave et al. investigated the reasons for postoperative dissatisfaction after primary THA. In 57 out of 78 patients they detected muscular weakness and were able to improve patient satisfaction by special physiotherapy [6]. This highlights the relevance of this study with isokinetic strength measurement in detail.

Furthermore, we imposed the coefficient of variation at every isokinetic measurement, representing a dimension of how accurate and reproducible each movement was performed. We detected a tendency towards lower values in the ERAS group, resembling a higher reproducibility and security in movement in comparison to the conventional group. This finding indicates, that patients in the ERAS group seem to rehabilitate faster than patients from the conventional group.

The same tendency was seen in the evaluation of positive Trendelenburg’s sign and patients’ ability for One-leg stand for longer than 15 s. We detected an increase in positive Trendelenburg’s sign and reduced patients’ ability for One-leg stand in the Non-ERAS group five days postoperatively, in comparison to the preoperative situation reaching better results four weeks after the operation. In the ERAS group this observation was much less pronounced for one-leg stand, while positive Trendelenburg’s sign was seen even lesser than preoperative already five days after the operation. Different studies already proved the advantages of an ERAS concept for total joint arthroplasty as there were less thromboembolic events or gastrointestinal adverse reactions [2, 16, 17]. The results of the present study support the implementation of an ERAS concept for primary THA. In contrast to different enhanced recovery after TJA studies, the present study does not focus on reduction of the length of hospital stay [27]. Due to general laws in the German health system (SGBV) the patients in both groups were discharged to the rehabilitation clinic five to seven days after the surgery took place.

While there is a lot of literature and proofs for Isokinetic strength measurement for the knee, there is much less for the hip joint. A lot of studies use a hand-held dynamometer to assess muscular strength of the hip. However, because of a weak interobserver reliability, influenced by testers strength and sex, this diagnostic tool is discussed quite controversially [29, 30]. Therefore, isokinetic dynamometers, as used in this study, represent the gold standard for a differentiated assessment of muscular strength [7]. The use of a Biodex system for isokinetic strength measurement of the hip is described in different studies and has shown a high reliability for flexion with an angular speed of 60°/s, as used in this study [11, 32]. Another study proved the reliability of the Biodex system for isokinetic hip abduction in children [32]. However, one must admit that hard reference values for isokinetic measurement of hip strength are still missing [10]. We used an experimental setup with patients standing in an upright position in front of the Biodex system. A recent systematic review in athletic patients proved a good reliability for measurement of hip flexion strength in a standing position. However, this setup requires identical positioning as well as fixed handlebars for patients’ safety, as our patient cohort met a mean age of 68 years. Mostly non-weight-bearing isokinetic measurement is performed for comparison between both legs [1, 23]. However, in comparison to an upward setup, a non-weight-bearing, lying setup is not able to reproduce physiological motion of daily life.

There are only a few studies which performed isokinetic strength measurement in patients with THA. A recent study compared two groups of patients who received THA. One group underwent intensive sport rehabilitation for one year postoperative, the other one served as a control. The authors performed strength measurement at baseline, six months, and twelve months postoperatively. At both follow-up time points, the authors could not show a significant benefit in isokinetic muscular strength after an intensive sport rehabilitation program. However, they detected a tendency towards better results in the intervention group [3]. One must admit that the authors did not evaluate the first weeks and months postoperatively and set the first follow-up time point rather late at six months. A different study compared the posterior and anterolateral approach for primary THA in terms of isokinetic strength measurement with a Biodex system. They did not detect a significant difference in muscular strength between both approaches after six and twelve months [9]. Tanaka et al. studied the influence of hip center position on abductor muscular strength with a Biodex system. They detected an association between superior placement and delayed recovery of abductor muscular strength [28]. Although total hip arthroplasty (THA) is expected to result in a postoperative loss of muscular strength, no study investigated the benefit of an ERAS concept on skeletal muscles surrounding the hip in detail.

One major disadvantage of the Biodex system is its time-consuming assessment. This may be one of the reasons why most publications involve a relatively small patient cohort. There is still potential for incorrect measurements, for example by wrong height positioning of the dynamometer. However, one must admit that the risk is much lower than with a handheld dynamometer. The biggest potential error in isokinetic strength measurement is the patient himself. The results strongly depend on patients’ motivation, pain, and individual feeling on that day.

The present study featured a high dropout rate of 22.5%. Almost half of the drop-out rate was caused by SARS-CoV-19 infections. Due to the SARS-CoV-19 pandemic, elective surgeries were generally halted in Germany for an extended period. All patients were tested for SARS-CoV-19 preoperatively. Three patients dropped out because of a newly diagnosed SARS-CoV-19 infection, and in those cases no operation was allowed for at least twelve weeks.

Limitations of the present study mainly represent the small sample size with twelve patients in each group. The present study is part of a big randomized controlled trial, however the measurement with the Biodex System is quite complex and time-consuming for the study crew but also for the patient himself. Consequently, it was not possible to include more patients. Moreover, though we used a Biodex system, the results still depend on patients’ motivation and daily health condition as described above. Strengths are a prospective, single-blinded randomized controlled trial with a highly standardized study protocol, the use of a Biodex system and multiple validated questionnaires.

## Conclusion

In a prospective, single-blinded randomized controlled trial, we proved a significant reduced loss of muscular strength five days as well as four weeks postoperatively after primary THA in combination with an ERAS concept (*p* < *0.05)*. However, the reduced loss of muscular strength is not reflected by patient’s functional outcome and quality of life, showing no significant differences in WOMAC, HHS, EQ-5d-3L, PROMs and NRS. Therefore, this study supports the implementation of an ERAS concept for primary THA with regards to isokinetic strength. Further studies are needed to evaluate the development of muscular strength over a longer postoperative period.

## Data Availability

On request, data is available at the authors’ institution.
